# Correction: Exposure to organochlorine pesticides as a predictor to breast cancer: A case-control study among Ethiopian women

**DOI:** 10.1371/journal.pone.0260106

**Published:** 2021-11-11

**Authors:** Seblework Mekonen, Mohammedgezali Ibrahim, Higemengist Astatkie, Aynalem Abreha

There are errors in the Funding statement. The correct Funding statement is as follows: The grant holder is Seblework Mekonen, Award agreement No = 4500384863. This work was carried out with aid of a grant from United Nations Educational, Scientific and Cultural Organization (UNESCO) and International Development Research Centre (IDRC), Ottawa, Canada. The views expressed herein do not necessarily represent UNESCO, IDRC or its board of governors. The funders had no role in study design, data collection and analysis, decision to publish, or preparation of the manuscript.
