# Supplementary material for: Exposure to organochlorine pesticides as a predictor to breast cancer: A case-control study among Ethiopian women
Source: PLoS One. 2021 Sep 23;16(9):e0257704. doi: 10.1371/journal.pone.0257704 (PMC8460037; doi:10.1371/journal.pone.0257704)
Supplement: S1 Data — (DOCX) [file pone.0257704.s001.docx]

**Data Collection tools**

# DATA COLLECTION TOOLS

JIMMA UNIVERSITY

INISTITUTE OF HEALTH

Faculty of public Health

Department of Environmental Health Science and Technology

Questionnaires for the assessment of breast cancer risk factors in association with exposure to organochlorine pesticides

**General information**

Code/ID: …………………………………………………………………………………………..

Address: ……………………………………………………………………………………………

1. **Socio demographic characteristics of respondents**
2. Age ………………………………………
3. What is your educational status?
   1. Illiterate b. read and write c. primary school d. secondary school e. tertiary school
4. Where is your residence?
   1. Rural b. semi-urban c. urban
5. What is your marital status?
   1. Married b. single c. divorced d. widowed
6. What is your religion?
   1. Orthodox b. Muslim c. protestant d. others (specify)
7. What is your occupation?
   1. Farmer b. merchant c. employee (Gov.t or non-gov.t) d. housewife f. other(specify)
8. What is your source of income?_______________

**II. Background of the study participants explaining biological and behavioral factors?**

1. Are you new or follow-up patient?
   1. Yes b. follow-up
2. How old were you when you began to menstruate? _____________
3. Was your menses regular at 18 years of age
   1. YES b. NO
4. Do you still have menses?
   1. YES b. NO
5. If NO, How old were you when menopause occurred? ____________
6. How many times have you been married?______________
7. At what age was your first marriage?
   1. YES b. NO
8. How many times have you been pregnant? (include live birth, still birth, miscarriages and abortions)---------------------------
9. How old were you, when you were pregnant for the first time? ------------
10. How many times have you given birth? (Include still births after
    8th month)?____________________
11. Did you breast-feed your child/children?
    1. YES b. NO
12. If yes, for question No 17, for how many children?
13. For how long did you breast-feed one child? ___________
14. Did you have miscarriages or abortions?
    1. YES b. NO
15. If yes, for question No 20 How many times_____________
16. Have you ever used Contraceptives?
    1. YES b. NO
17. If yes, for question No 22 for how long _____________
18. What types of contraceptives you use?
    1. Injection b. OCP C. Implant d. Others (specify )__________
19. What type of food you ate commonly?
    1. Animal products (eg. Milk, butter, meat, egg etc)…………………………………
    2. Plants products (eg. Vegetable like tomato, potato, cabbage, etc)_______________
20. Did someone in a family diagnosed for breast/ovarian cancer?
    1. YES b. NO
21. If yes for question No 26 which family member has the history?
    1. Mother b. father C. sister D. grandmother E. brother f. aunt
22. Have you had a breast biopsy? For control only
    1. YES b. NO
23. Do/did you smoke? / Does/did someone smoke inside your living house?

a. YES b. NO

1. If yes, how many cigarettes do you smoke per day?
2. How many hours per day do/did you spend in cigarette smoke-filled apartment (estimate)? _________
3. Does/did someone smoke at your workplace?

. YES b. NO

1. Do/did you drink alcoholic beverages and what type? E.g. Tella, bear, ---------?

a. YES b. NO

1. How old were you, when you start drinking alcohol habitually?________

**III. Environmental and occupational factors associated with BC etiology**

1. Do you know what chemical pesticides are?
   1. Yes b. No
2. Have you ever use/used any chemical pesticides?
   1. Yes b. No
3. If yes, where?
   1. In living house b. farming areas c. working palace d. garden
4. Who were applying chemical pesticides from your family?
   1. Father b. mother c. others (specify)_________
5. Where do/did you or the family keep pesticides?
   1. Roof b. under bed c. together with food on shelf d. outside home e. others (specify)___________
6. Do you have any experience of mixing-loading/ application of pesticides? \
   1. Yes b. No
7. Where you or the families do/did put the pesticide containers after used?
   1. Open field b. use it at household c. throw to the river d. others (specify)_______
8. Is there a flower industry near to your living/working area?( estimate the distance)__________
   1. Yes b. No
9. Do you ever worked in flower industry?
   1. Yes b. No
10. If yes, for question No 44, for how long (years)? ____________
    1. Yes b. No
11. Have you been washed the food items (wheat, vegetables, pea or bean, fruits and others) before changing to flour/ use? Using water or what?
    1. Yes b. No
12. If yes do/did you/your family use insecticides?
    1. Yes b. No
13. From where you get chemical pesticides?
    1. Shops b. illegal sellers c. farmers association
14. If question no 48 is yes how do/did you/your family apply insecticides

A using spraying equipment b, using traditional method (using plant leaf, cloth, cotton) c. others (Spesify)

1. How often do/did you apply?
   1. Weekly, b. Monthly c annually d, other (specify)________
2. Do/did you/your family use Personal rotective equipment during application of insecticides?
   1. Yes b. No
3. What is your drinking water source?
   1. Ground water b. surface water
4. Have you/your families ever participate in farming?
   1. Yes b. No
5. If question no 53 is yes, do/did you/your families apply pesticides?
   1. Yes b. No
6. If question no 54 is yes, for how long? _____________________
   1. From where do/did you buy chemical pesticides Shops b. open market merchants c. farmers associations d. others specify _______
