# Supplementary material for: Exposure to organochlorine pesticides as a predictor to breast cancer: A case-control study among Ethiopian women
Source: PLoS One. 2021 Sep 23;16(9):e0257704. doi: 10.1371/journal.pone.0257704 (PMC8460037; doi:10.1371/journal.pone.0257704)
Supplement: S1 Questionnaire — (DOCX) [file pone.0257704.s003.docx]

**ጅማ ዩኒቨርሲቲ**

**ጤና ሳይንስ ኢንስቲትዩት**

**የህብረተሰብ ጤና ፋካልቲ**

**የአካባቢ ጤና አጠባበቅ ሳይንስና ቴክኖሎጂ ት/ት ክፍል**

ከኦርጋኖክሎሪን ፀረ-ተባይ ኬሚካሎች ተጋላጫነት ጋር ተያይዘው ለሚመጡ የጡት ካንሰር ጋር ችግሮች ዳሰሳ መጠይቅ

**አጠቃላይ መረጃ**

የተሳታፊ መለያ ቁጥር _________________

እድራሻ ______________________

1. **የመላሾች ማኅበራዊና ስነህዝብ ባህሪያት**
2. እድሜ ­­­-------------------
3. የትምህርት ደረጃዎ

ሀ.ያልተማረ ለ. ማንበብና መፃፍ ሐ. የመጀመሪያ ደረጃ ትምህርት መ. ሁለተኛ ደረጃ ትምህርት ሠ. የከፍተኛ ትምህርት ረ. ሌላ ካለ ይግለጽ__________

1. የመኖሪያዎ ቦታ የት ነው?

ሀ. ገጠር ለ.የገጠር ከተማ ሐ.ከተማ

1. የጋብቻ ሁኔታ

ሀ. ያገባች ለ.ያላገባች ሐ.የፈታች መ. በሏ የሞተ/ያረፈ

1. ሀይማኖት

ሀ. ኦርቶዶክስ ለ.ሙስሊም ሐ.ፐሮቴስታንት መ.ሌላ (ይግለጹ)________________

1. የስራ ሁኔታ

ሀ. አርሶ አደር ለ. ነጋዴ ሐ. ተቀጣሪ(መንግስታዊ / መንግስታዊ ባልሆነ ተቋም) መ. የቤት እመቤት ረ. ሌላ (ይግለጹ)_______________

1. የገቢ ምንጭዎ ምንድነዉ?____________________________________________
2. **የተሳታዎች ተፈጥሯዊና ባህሪያዊ ሁኔታ**
3. ተከታታይ ታካሚ ኖት ወይስ አዲስ

ሀ. አዲስ ለ.ተከታታይ

1. የወር አበባ ማየት የጀመርሽው በስንት አመትሽ ነበር________________
2. በ18 አመትሽ የወር አበባሽ በግዜው(በትክክለኛው ጊዜ) ታያለሽ

ሀ. አዎ ለ. አይ

1. አሁንም የወር አበባ ያያሉ

ሀ. አዎ ለ. አይ

1. የ ተ.ቁ11. ምላሽ አይ ከሆነ እድሜዎት ስንት ሲሆን ማየት አቆሙ___________
2. አግብተሻል? ሀ. አዎ ለ. አይ
3. ስንት ግዜ?……………
4. የመጀመሪያ ጋብቻዎን በስንት አመት ነበር ያከናወኑት________
5. ለ ስንት ጊዜ አርግዘው ያውቃሉ_______________
6. የመጀመሪያ ልጅዎን ሲያረግዙ እድሜዎ ስንት ነበር?_________
7. ልጆቸዎን ጡት ያጠባሉ

ሀ. አዎ ለ. አይ

1. የጥያቄ ቁጥር.18. ምላሽ አዎ ከሆነ ፤ ለምን ያህል ጊዜ ያጠባሉ?__________
2. እርግዝና ተሳናክሎቦት ያውቃል

ሀ. አዎ ለ. አይ

1. የ ጥያቄ ቁጥር.20. ምላሽ አዎ ከሆነ ፤ ምን ያህል ጊዜ____________
2. የወሊድ መቆጣጠሪያ ወስደው ያውቃሉ?

ሀ. አዎ ለ. አይ

1. የጥያቄ ቁጥር.22. ምላሽ አዎ ከሆነ ፤ ለምን ያህል ጊዜ_______________
2. ምን አይነት የወሊድ መቆጣጠሪያ

ሀ. መርፌ ለ.ኪኒኒ ሐ.በእጅ የሚቀበር መ.ሌላ(ይግለፁ)_______________

1. በአብዛኛውን የሚጠቀሙት ምን አይነት ምግብ ነው?

ሀ.የአንሰሳት ተዋፅዎ፡ ለምሳሌ ወተት፣ እንቁላል፣ ስጋ _________

ለ. የእጽዋት ውጤቶች፡ ጤፍ እንጀራ፣ ቲማቲም፣ ድንች______________

1. ከቤተሰብዎ አባል የጡት ካንሰር ህመምተኛ አለ

ሀ. አዎ ለ. አይ

1. ጥያቄ ቁጥር.26 ምላሽ አዎ ከሆነ ፤ የትኛው የቤተሰብ አባል?

ሀ. እናት ለ. አባት ሐ. እህት መ . ወንድም ሠ. አክስት ረ. የሴት አያት

1. ለጡት ካንሰር ተመርምረው ያውቃሉ?(control)

ሀ. አዎ ለ. አይ

1. ሲጋራ ያጫሳሉ/እቤትዎ ውስጥ የሚያጨስ አለ

ሀ. አዎ ለ. አይ

1. ጥያቄ ቁጥር.29. ምላሽ አዎ ከሆነ ፤በቀን ምን ያህል ሲጋራ (በግምት)____________
2. የሲጋራ ጪስ ባለበት ቤት ውስጥ በቀን በግምት ለምን ያህል ጊዜ ይቆያሉ__________
3. በስራ ቦታዎ የሚያጨስ አለ

ሀ. አዎ ለ. አይ

1. የአልኮል መጠጥ ይጠጣሉ? ሀ. አዎ ለ. አይ
2. አዎ ካሉ፣ በስንት አመት መጠጣት ጀመሩ?
3. የትኛውን የአልኮል መጠጥ የጠጣሉ? ምሳሌ ቢራ፡ ጠላ____________
4. **ከጡት ካንሰር ጋር ተያዥነት ያለው አካባቢያዊ እና የስራ ሁኔታዎች**
5. ፀረ-ተባይ ኬሚካል ስንል ምን እንደሆነ ያውቃሉ?

ሀ. አዎ ለ. አይ

1. እርሶ/(የቤተሰብዎ አባል) ፀረ-ተባይ ኬሚካል ተጠቅመው ያውቃሉ

ሀ. አዎ ለ. አይ

1. ጥያቄ ቁጥር..2. ምላሽ አዎ ከሆነ የት ነው የሚጠቀሙት?

ሀ.በመኖሪያ ቤት ለ.በእርሻ ቦታ ሐ.በስራ ቦታ መ.ቤት ውስጥ

ሠ.ግቢ ዉስጥ ረ. ሌላ(ይግለጹ)…..…..

1. ጥያቄ ቁጥር 2. ምላሽ አዎ ከሆነ ከቤተሰብዎ ማነው የሚረጨው?

ሀ. እናት ለ. አባት ሐ. እህት መ . ወንድም ሠ.ባለቤት/የትዳር አጋር

1. እርሶዎ/(የቤተሰብዎ አባል) ፀረ-ተባይ ኬሚካል የት ያስቀምጣሉ?

ሀ.ጣራ ላይ ለ. አልጋ ስር ሐ.መደርደሪያ ላይ ከምግብ ጋር መ.ሌላ(ይግለጹ)_________________

1. ፀረ-ተባይ ኬሚካልን የመደባለቅና የመርጨት ልምድ አለዎት

ሀ. አዎ ለ. አይ

1. እርሶዎ /(የቤተሰብዎ አባል) የፀረ-ተባይ ኬሚካል አቃዎችን የት የጥላሉ?

ሀ.ሜዳ ላይ ለ. ለቤት ውስጥ መገልገያ አውለዋለው ሐ. ወንዝ መ. ሌላ(ይግለጹ)…………………

1. በመኖሪያ አካባቢዎ የአበባ እርሻ አለ( ርቀት በግምት)________________

ሀ. አዎ ለ. አይ

1. በአበባ እርሻ ውስጥ ሰርተው ያውቃሉ?

ሀ. አዎ ለ. አይ

1. ጥያቄ ቁጥር 38 ምላሽ አዎ ከሆነ ለምን ያህል ጊዜ_______________
2. የእህል ዘሮችን ለምሳሌ ጤፍ ፡በርበሬ፤ ስንዴ ፣ የመሳሰሉትን ከማሰፈጨትዎ በፊት ያጥባሉ? በዉሀ ወይስ በምን?

ሀ. አዎ ለ. አይ

1. እርሶዎ/(የቤተሰብዎ አባል) ፀረ-ነፍሳት ኬሚካል ይጠቀማሉ

ሀ. አዎ ለ. አይ

1. ጥያቄ ቁጥር. 41. ምላሽ አዎ ከሆነ እንዴት ይጠቀማሉ

ሀ.ለዚው ተግባር በተዘጋጀ መርጫ እቃ ለ. በተለምዶ(በጭራ፡በልብስ ……)

1. ምን ያህል ጊዜ ይጠቀማሉ

ሀ.በሳምነት ለ.በወር ሐ.በአመት መ.ሌላ(ይግለጹ)…………………

1. እርሶዎ/የቤተሰብዎ አባል ፀረ-ነፍሳት ኬሚካል ሲጠቀሙ የራስ መከላከያ ልብስ (PPE) ይጠቀማሉ

ሀ. አዎ ለ. አይ

1. የመጠጥ ዉሀ ከየት ያገኛሉ

ሀ.የከርሰ ምድር ውሀ ለ.ከወንዝ ሐ.የቧንቧ ውሀ መ.ሀይላንድ

1. የግብርና ስራ ላይ ተሰማርተው ያውቃሉ

ሀ. አዎ ለ. አይ

1. ጥያቄ ቁጥር 46. ምላሽ አዎ ከሆነ ፀረ-ተባይ ኬሚካል ተጠቅመው ያውቃሉ

ሀ. አዎ ለ. አይ

1. ጥያቄ ቁጥር 47. ምላሽ አዎ ከሆነ ለምን ያህል ጊዜ_________
   1. በመት ለ. በሳምንት ሐ. በወር መ. ሌላ (ይገለጽ)
2. ስለ ጸረ ተባይ ስልጠና ወስደዉ ያዉቃሉ

ሀ. አዎ ለ. አይ

1. ፀረ - ተባይ ኬሚካል ከየት ይገዛሉ?

ሀ. ከሱቅ ለ. ከ ነጋዴ ወይም ከ ገበያ ሐ. ከገበሬ ማህበር መ. ሌላ (ይገለጽ)
