# Supplementary material for: Exposure to organochlorine pesticides as a predictor to breast cancer: A case-control study among Ethiopian women
Source: PLoS One. 2021 Sep 23;16(9):e0257704. doi: 10.1371/journal.pone.0257704 (PMC8460037; doi:10.1371/journal.pone.0257704)
Supplement: Attachment — Submitted filename: Response to Reviewers.docx [file pone.0257704.s004.docx]

**Response to Reviewers**

***Dear Editor and reviewers of PLOS ONE journal, the authors are very much grateful for giving us the opportunities to review and submit the revised version of our manuscript and for your scholarly comments. All the comments are addressed in the manuscript and the major changes are shown in track change. Here under, please kindly accept our response to your comments.***

PONE-D-21-17256
Exposure to organochlorine pesticides as a predictor to breast cancer: a case-control study among Ethiopian women
PLOS ONE

Journal Requirements:

When submitting your revision, we need you to address these additional requirements.

1. Please ensure that your manuscript meets PLOS ONE's style requirements, including those for file naming. The PLOS ONE style templates can be found at
   https://journals.plos.org/plosone/s/file?id=wjVg/PLOSOne_formatting_sample_main_body.pdf and 
   <https://journals.plos.org/plosone/s/file?id=ba62/PLOSOne_formatting_sample_title_authors_affiliations.pdf>

***Thank you for your comment. The manuscript is corrected according to PLOS ONE style requirements.***

2. Please include additional information regarding the survey or questionnaire used in the study and ensure that you have provided sufficient details that others could replicate the analyses. For instance, if you developed a questionnaire as part of this study and it is not under a copyright more restrictive than CC-BY, please include a copy, in both the original language and English, as Supporting Information.

***Thank you for your comment. Questionnaires in both original language and English we were prepared and used for the survey part of this manuscript is submitted as supplementary document***

3. Thank you for stating the following in the Acknowledgments Section of your manuscript:

We are highly indebted to the Organization for Women in Science for The Developing World (OWSD) Secretariat for their unreserved support and follow-up of the proper running of the project. This study was carried out by the aid of a grant obtained from UNESCO and International Development Research Cntre (IDRC) Ottawa Canada, and the authors deeply acknowledged the funders.

Please note that funding information should not appear in the Acknowledgments section or other areas of your manuscript. We will only publish funding information present in the Funding Statement section of the online submission form.

***Thank you for your critical comment. The funding information is removed from the acknowledgment part and mentioned in the funding statement on page 18.***

Please remove any funding-related text from the manuscript and let us know how you would like to update your Funding Statement. Currently, your Funding Statement reads as follows:

The grant holder is Seblework Mekonen,
The study was funded by a grant obtained from UNESCO/IDRC under the Organization for Women in Science for The Developing World (OWSD), Award agreement No.: 4500384863
The funders had no role in study design, data collection and analysis, decision to publish, or preparation of the manuscript

Please include your amended statements within your cover letter; we will change the online submission form on your behalf.

The amended funding statement is included in the cover letter.

***Thank you for the critical comment you gave us. The statement acknowledged the funding organization is removed from the acknowledgment part in the manuscript and is addressed in the funding statement as well as included in the cover letter to amend in the online submission.***

4. We note that you have indicated that data from this study are available upon request. PLOS only allows data to be available upon request if there are legal or ethical restrictions on sharing data publicly. For more information on unacceptable data access restrictions, please see http://journals.plos.org/plosone/s/data-availability#loc-unacceptable-data-access-restrictions.

In your revised cover letter, please address the following prompts:

a) If there are ethical or legal restrictions on sharing a de-identified data set, please explain them in detail (e.g., data contain potentially sensitive information, data are owned by a third-party organization, etc.) and who has imposed them (e.g., an ethics committee). Please also provide contact information for a data access committee, ethics committee, or other institutional body to which data requests may be sent.

b) If there are no restrictions, please upload the minimal anonymized data set necessary to replicate your study findings as either Supporting Information files or to a stable, public repository and provide us with the relevant URLs, DOIs, or accession numbers. For a list of acceptable repositories, please see <http://journals.plos.org/plosone/s/data-availability#loc-recommended-repositories>.

***Thank you for your concern. There is no legal restriction and the data is submitted as by using the link for data repositories.***

We will update your Data Availability statement on your behalf to reflect the information you provide.

5. PLOS requires an ORCID iD for the corresponding author in Editorial Manager on papers submitted after December 6th, 2016. Please ensure that you have an ORCID iD and that it is validated in Editorial Manager. To do this, go to ‘Update my Information’ (in the upper left-hand corner of the main menu), and click on the Fetch/Validate link next to the ORCID field. This will take you to the ORCID site and allow you to create a new iD or authenticate a pre-existing iD in Editorial Manager. Please see the following video for instructions on linking an ORCID iD to your Editorial Manager account: <https://www.youtube.com/watch?v=_xcclfuvtxQ>

***Thank you. This is addressed.***

6. We note that Figure 1 in your submission contain [map/satellite] images which may be copyrighted. All PLOS content is published under the Creative Commons Attribution License (CC BY 4.0), which means that the manuscript, images, and Supporting Information files will be freely available online, and any third party is permitted to access, download, copy, distribute, and use these materials in any way, even commercially, with proper attribution. For these reasons, we cannot publish previously copyrighted maps or satellite images created using proprietary data, such as Google software (Google Maps, Street View, and Earth). For more information, see our copyright guidelines: <http://journals.plos.org/plosone/s/licenses-and-copyright>. 
We require you to either (1) present written permission from the copyright holder to publish these figures specifically under the CC BY 4.0 license, or (2) remove the figures from your submission:
 
a. You may seek permission from the original copyright holder of Figure 1 to publish the content specifically under the CC BY 4.0 license.  
 
We recommend that you contact the original copyright holder with the Content Permission Form (http://journals.plos.org/plosone/s/file?id=7c09/content-permission-form.pdf) and the following text:
“I request permission for the open-access journal PLOS ONE to publish XXX under the Creative Commons Attribution License (CCAL) CC BY 4.0 (http://creativecommons.org/licenses/by/4.0/). Please be aware that this license allows unrestricted use and distribution, even commercially, by third parties. Please reply and provide explicit written permission to publish XXX under a CC BY license and complete the attached form.”
 
Please upload the completed Content Permission Form or other proof of granted permissions as an "Other" file with your submission.
 
In the figure caption of the copyrighted figure, please include the following text: “Reprinted from [ref] under a CC BY license, with permission from [name of publisher], original copyright [original copyright year].”
 
b. If you are unable to obtain permission from the original copyright holder to publish these figures under the CC BY 4.0 license or if the copyright holder’s requirements are incompatible with the CC BY 4.0 license, please either i) remove the figure or ii) supply a replacement figure that complies with the CC BY 4.0 license. Please check copyright information on all replacement figures and update the figure caption with source information. If applicable, please specify in the figure caption text when a figure is similar but not identical to the original image and is therefore for illustrative purposes only.
The following resources for replacing copyrighted map figures may be helpful:
 
USGS National Map Viewer (public domain): http://viewer.nationalmap.gov/viewer/
The Gateway to Astronaut Photography of Earth (public domain): http://eol.jsc.nasa.gov/sseop/clickmap/
Maps at the CIA (public domain): https://www.cia.gov/library/publications/the-world-factbook/index.html and https://www.cia.gov/library/publications/cia-maps-publications/index.html
NASA Earth Observatory (public domain): http://earthobservatory.nasa.gov/
Landsat: http://landsat.visibleearth.nasa.gov/
USGS EROS (Earth Resources Observatory and Science (EROS) Center) (public domain): http://eros.usgs.gov/#
Natural Earth (public domain): http://www.naturalearthdata.com/

***Thank you for your comment. The map in Fig.1 was constructed by the authors of this manuscript using ArcGIS software v. 10.3.***

Please review your reference list to ensure that it is complete and correct. If you have cited papers that have been retracted, please include the rationale for doing so in the manuscript text, or remove these references and replace them with relevant current references. Any changes to the reference list should be mentioned in the rebuttal letter that accompanies your revised manuscript. If you need to cite a retracted article, indicate the article’s retracted status in the References list and also include a citation and full reference for the retraction notice.

***Thank you for your critical view and comments. All the references are checked and there is no retracted reference sited in our manuscript.***

Additional Editor Comments (if provided):

The manuscript require a minor revision for publication.

***Thank you very much for all you critical, detail comments and suggesting minor revision***.

[Note: HTML markup is below. Please do not edit.]

Reviewers' comments:

Reviewer's Responses to Questions

**Comments to the Author**

1. Is the manuscript technically sound, and do the data support the conclusions?

The manuscript must describe a technically sound piece of scientific research with data that supports the conclusions. Experiments must have been conducted rigorously, with appropriate controls, replication, and sample sizes. The conclusions must be drawn appropriately based on the data presented.

Reviewer #1: Yes

Reviewer #2: Yes

2. Has the statistical analysis been performed appropriately and rigorously?

Reviewer #1: Yes

Reviewer #2: Yes

3. Have the authors made all data underlying the findings in their manuscript fully available?

The [PLOS Data policy](http://www.plosone.org/static/policies.action#sharing) requires authors to make all data underlying the findings described in their manuscript fully available without restriction, with rare exception (please refer to the Data Availability Statement in the manuscript PDF file). The data should be provided as part of the manuscript or its supporting information, or deposited to a public repository. For example, in addition to summary statistics, the data points behind means, medians and variance measures should be available. If there are restrictions on publicly sharing data—e.g. participant privacy or use of data from a third party—those must be specified.

Reviewer #1: Yes

Reviewer #2: No

4. Is the manuscript presented in an intelligible fashion and written in standard English?

PLOS ONE does not copyedit accepted manuscripts, so the language in submitted articles must be clear, correct, and unambiguous. Any typographical or grammatical errors should be corrected at revision, so please note any specific errors here.

Reviewer #1: Yes

Reviewer #2: Yes

5. Review Comments to the Author

Please use the space provided to explain your answers to the questions above. You may also include additional comments for the author, including concerns about dual publication, research ethics, or publication ethics. (Please upload your review as an attachment if it exceeds 20,000 characters)

Reviewer #1: This paper investigates the role of organochlorine pesticide as a predictor of breast cancer. In the study, authors have collected samples and analyzed for the pesticide levels. In addition, they have also conducted survey of the participants. Further, detailed statistical analyses are done to correlate the presence of pesticide to the breast cancer. The study is well designed. In addition, comprehensive analyses are performed and reported. I recommend that this manuscript could be accepted with minor revision. There are few issues which can be addressed, as mentioned below:
1. The language could be improved since there are many spelling and grammatical mistakes throughout the manuscript.

***Thank you very much for your critical and positive comments. The spelling and grammatical mistakes are corrected in the manuscript***

2. References are not properly cited - see 2nd line in introduction. Also, multiple reference styles are used - see page 6, second line.

***Thank you for your comment. References are corrected throughout the manuscript according to the journal requirement. The style is corrected to Vancouver reference style****.*

3. Provide full form at first occurrence - OCP in last paragraph of introduction.

***Thank you for your comment. This is addressed on page 4 last paragraph of introduction.***

Reviewer #2: The article highlights a very important environmental issue. However, there has to be some revisions before acceptance

***Authors are very thankful for your critical view and positive comments.***

Page 3: Please rephrase the last sentence “The remaining unidentified causes continue to interest the scientific communities to give attention to…”

***Thank you. This is addressed on page 3.***

Please spell check the manuscript

***Thank you. This is addressed throughout the manuscript.***

Please use units uniformly.

***Thank you. All addressed in the manuscript***

Section 3.1: Report values only up to significant decimal values

***Thank you. Comment addressed in the manuscript***

In the abstract it says data was collected for 100 people, however the total comes out only to be 50.

***Thank you. This is addressed in the abstract on page 2. Blood samples were collected from 100 participants (50 from breast cancer patients and 50 from controls****.*

Please grammar check the manuscript

***Thank you and all are corrected throughout the manuscript****.*

For logistic regression please explain it terms of response and independent variables

***Thank you. Comments addressed under sub section of data bases and statistical analysis and results explanations***

Make sure that the independent variables are mutually exclusive, may not be in some cases
More detailed explanations of OR significance required, what does it mean it terms of behavior of the response

*Dependent variable as response (outcome) variable was mutually exclusive mean the response is either they develop breast cancer or not develop breast cancer. There is no way for the responses to be both simultaneously. For the predictor (independent variable) we have tried to look at the effect of the coincidences of the variables by multicollinearity among these independent variables weather they correlated to each other instead just to response variable.*
Each acronym has to be expanded when first used

***Thank you and all addressed in the manuscript according to your comment***

6. PLOS authors have the option to publish the peer review history of their article ([what does this mean?](https://journals.plos.org/plosone/s/editorial-and-peer-review-process#loc-peer-review-history)). If published, this will include your full peer review and any attached files.


If you choose “no”, your identity will remain anonymous but your review may still be made public.


**Do you want your identity to be public for this peer review?** For information about this choice, including consent withdrawal, please see our [Privacy Policy](https://www.plos.org/privacy-policy).

Reviewer #1: No

Reviewer #2: No

 [NOTE: If reviewer comments were submitted as an attachment file, they will be attached to this email and accessible via the submission site. Please log into your account, locate the manuscript record, and check for the action link "View Attachments". If this link does not appear, there are no attachment files.]

While revising your submission, please upload your figure files to the Preflight Analysis and Conversion Engine (PACE) digital diagnostic tool, <https://pacev2.apexcovantage.com/>. PACE helps ensure that figures meet PLOS requirements. To use PACE, you must first register as a user. Registration is free. Then, login and navigate to the UPLOAD tab, where you will find detailed instructions on how to use the tool. If you encounter any issues or have any questions when using PACE, please email PLOS at [figures@plos.org](mailto:figures@plos.org). Please note that Supporting Information files do not need this step
